# Experiences of Advanced Non-Small Cell Lung Cancer Patients with Targeted Therapy Using Journey Mapping: A Qualitative Study

**DOI:** 10.3390/curroncol32080451

**Published:** 2025-08-11

**Authors:** Hailing Tu, Minghui Wang, Shengmei Yang, Jingfang Hong

**Affiliations:** School of Nursing, Anhui Medical University, 81# Meishan Road, Hefei 230032, China; thl18755930259@163.com (H.T.); wangminghui202204@163.com (M.W.); 15761425775@163.com (S.Y.)

**Keywords:** advanced non-small cell lung cancer, patient journey mapping, precision medicine, treatment experience, qualitative interview

## Abstract

Targeted therapies against specific driver gene mutations have become the standard first-line treatment for most patients with advanced non-small cell lung cancer (NSCLC). While these therapies significantly prolong survival, the entire cancer treatment journey remains challenging and distressing. To better understand these experiences, this study employed a qualitative descriptive approach, conducting semi-structured interviews with 18 advanced NSCLC patients receiving targeted therapy, supplemented by patient journey logs. The resulting journey map delineated five stages: diagnosis, initial treatment, maintenance therapy, disease progression, and end-of-life. The analysis identified four key themes characterizing patient experiences at each stage. These findings enable healthcare professionals to identify risk situations and determine optimal timing for support interventions. Similarly, preparing patients for the processes they must undergo and the side effects of medical treatment helps reduce their uncertainty and anxiety, thereby improving their quality of life.

## Simple Summary

Targeted therapy has become the standard treatment for advanced NSCLC patients with specific driver gene mutations, significantly improving survival outcomes. However, the entire cancer treatment journey still faces numerous challenges. This study aims to explore the whole disease experience of patients receiving targeted therapy through a patient journey mapping approach, thereby providing recommendations for clinical care practices. We invited 18 patients with advanced NSCLC receiving targeted therapy to share their experiences and invited clinical experts, oncology nurses, and patients to validate the journey map. Patients reported that their treatment journey was stage-specific, primarily characterized by ongoing financial burdens, dynamic psychological stress, and symptom-related and life challenges during disease progression. Notably, current medical support often fails to address these core needs sufficiently. The study results highlight key features and challenges at each stage of the disease, emphasizing the importance of clinicians providing personalized interventions at critical treatment milestones.

## 1. Introduction

Advanced non-small cell lung cancer (NSCLC) has historically had a poor prognosis, with a 5-year overall survival rate of approximately 6% [1]. However, recent advancements in genetic testing and targeted therapies have significantly improved treatment outcomes. Molecular-targeted drugs, such as EGFR and ALK inhibitors, have become the standard first-line treatments for advanced NSCLC patients with specific driver gene mutations, notably enhancing patient survival rates [2]. For instance, the 5-year overall survival rate for patients with advanced NSCLC who have anaplastic lymphoma kinase translocations and receive first-line alectinib is 62.5% [3].

Although targeted therapy offers hope for more prolonged survival, the overall process of cancer treatment can be difficult and distressing. Once treatment starts, patients must take oral targeted medications at home for extended periods, often dealing with side effects such as skin rashes, diarrhea, and oral ulcers on their own [4]. These side effects, combined with uncertainty about their prognosis, can lead to ongoing negative emotions including anxiety, depression, and fear, which significantly affect their quality of life and adherence to treatment [5,6]. Long-term survival with cancer also exposes patients to challenges like occupational instability, follow-up anxiety, and recurrence fears [7]. As treatment continues, the high cost of targeted drugs places a heavy financial burden on many families [8] Supporting and accompanying advanced NSCLC patients throughout this process requires a deep understanding of their experiences. Most existing studies have primarily focused on the patients’ experiences with targeted drug therapy [6] and the quality of life among long-term survivors [7,9]. Sanson-Fisher et al. [10] concluded that concentrating solely on specific aspects of patients’ experiences fails to address patients’ genuine needs; a comprehensive assessment of experiences across all care stages and the entire disease course remains crucial. Currently, there is a lack of research exploring the experiences of patients with advanced NSCLC who receive targeted therapy throughout the whole disease course and presenting it as a journey map.

Journey maps originated in the field of service design, and clinical researchers have adapted methods from marketing for use in clinical research, resulting in the development of patient journey maps [11]. The presentation forms of patient journey maps can be categorized into five types: Mental (Cognitive) Model Map, Experience Map, Customer Journey Map, Service Blueprint Map, and Spatial Map [12,13]. Among these, the Experience Map presents the end-to-end human experience sequentially and visually [14], illustrating patients’ behaviors, feelings, and needs at different stages of their healthcare journey [15]. This helps identify key touchpoints that can improve patient experiences.

By using this mapping approach, healthcare professionals can gain a deeper understanding of patients’ experiences throughout the healthcare process and provide targeted support at critical points [16]. This is significant for optimizing healthcare services’ quality and developing patient-centered care plans. Consequently, this study aims to construct an Experience Map for patients with advanced NSCLC receiving targeted therapy, identify potential concerns and gaps in care, and provide evidence to enhance the quality of healthcare services.

## 2. Materials and Methods

### 2.1. Ethical Considerations

The Bioethics Review Committee of Anhui Medical University (81250545) granted ethical approval. All participants signed informed consent forms.

### 2.2. Study Design

A qualitative descriptive design was adopted to achieve the research aim. The study adhered to the Standards for Qualitative Research Reports (SRQR) guidelines [17]. 

### 2.3. Participant Recruitment

This study was conducted at a tertiary general hospital in Hefei, Anhui Province, China. To comprehensively describe the patient experience, participants were recruited through purposive sampling from medical oncology, respiratory medicine, and radiation oncology departments based on characteristics such as gender, age, educational attainment, and treatment stage.

A systematic review of electronic medical records identified potential participants who met the eligibility criteria. Eligible patients were then provided with study information and enrollment invitations. The sample size was determined by data saturation, with recruitment ceasing when interview data began to repeat and no new themes emerged [18].

### 2.4. Inclusion and Exclusion Criteria

The inclusion criteria were as follows: (1) age ≥ 18 years; (2) pathologically confirmed unresectable advanced NSCLC, TNM stage IIIB-IV [1]; (3) received targeted therapy for ≥6 months; (4) awareness of their disease status; and (5) the ability to communicate effectively. Exclusion criteria included (1) a combination of other primary malignant tumors; and (2) participation in other interventional clinical trials.

A total of 7 patients declined the interview for various reasons: reluctance to discuss cancer-related topics due to psychological distress (3 patients), feeling physically weak (2 patients), or finding the process too time-consuming (2 patients).

### 2.5. Data Collection

A semi-structured interview guide was developed using a literature review and expert consultation (Table 1). A patient journey log was designed through research group discussions, including sections for recording key medical events, symptoms, contacts, and psychological changes and a free-writing section to provide a comprehensive understanding of the patient’s experience (Supplementary File S1). Before formal implementation, two pilot interviews were conducted to refine the interview guide and verify the operational feasibility of the journey log based on participants’ feedback.

The research team conducted face-to-face semi-structured interviews between December 2024 and March 2025, lasting between 40 and 60 min. The interviews were conducted in a private ward or a meeting room, depending on participants’ preferences. All interviews were conducted by a researcher (H. Tu) who had received training in qualitative research. The interviews were fully recorded, and non-verbal information, such as patients’ expressions, movements, and tone of voice, was documented.

Patient journey logs were used to document the patient experience throughout the entire hospitalization period continuously. On the day of admission, researchers distributed the logs and provided detailed instructions regarding their content, recording methods, and the scheduled timeframe for returns. Participants were encouraged to make entries as soon as possible after experiencing relevant events, thereby capturing immediate impressions. Family members assisted with mobile phone recordings for three patients who had not received education as an alternative to document key times and experiences. A researcher (S. Yang) continuously monitored recording progress and integrity through daily ward visits. Entries from the previous day were reviewed and collected the following morning. Any entries with ambiguous or unclear descriptions were promptly clarified through direct communication with the patient. After collection, researchers verified the events recorded in the logs by cross-referencing the entries with electronic medical records and consulting with the responsible nurses.

### 2.6. Data Analysis

Data collection and analysis took place simultaneously. Within 24 h of the interview, two researchers (H. Tu and M. Wang) transcribed the recordings verbatim, and a third researcher (S. Yang) verified the text data. Two researchers (H. Tu and M. Wang) conducted data analysis using NVivo 12 through the method of reflexive thematic analysis in an inductive and iterative way [19,20]. After data familiarization, the text was systematically coded based on the minimal meaningful units relevant to the main research question. Similar codes were then grouped to form initial, tentative themes (e.g., “selling a house” and “borrowing money” were categorized as “savings depletion”, while “savings depletion” and “treatment compromises” were categorized as “financial toxicity and economic impact”). The research team developed and refined these themes from three perspectives: as meaningful units in their own right, in terms of their interrelationships, and in relation to how they collectively shaped the overall story [20]. To establish the trustworthiness of the study, weekly discussions were held among the research team to review and align interpretations throughout the coding and analysis process.

Two researchers (H. Tu and M. Wang) analyzed the patient journey logs. The analysis specifically examined the associations between critical medical events (e.g., waiting for genetic testing, tumor recurrence, or metastasis) and corresponding emotional and behavioral responses, while also identifying additional experiences not mentioned during interviews for integration into the patient journey maps. The visualization of the patient journey maps was collaboratively performed by a graduate researcher (S. Yang) and the corresponding author (J. Hong).

### 2.7. Trustworthiness and Reflexivity

The research team comprised an oncology psychology expert and three nursing graduate students with backgrounds in qualitative research and oncology nursing. Throughout data collection and especially during analysis, we remained reflexively aware of how our professional roles and prior clinical experiences shaped our engagement with participants and interpretation of the data. We acknowledged that our subjectivity could enrich the analytic process and engaged in ongoing reflection on our positionality to ensure that interpretations were grounded in the data rather than overly influenced by prior assumptions or experiences [20].

Before the study, we developed an initial framework for the patient journey map through desk research and participatory observation. Data triangulation was employed during data analysis to integrate and cross-validate interview data, patient journey logs, and electronic medical records. The accuracy of the journey map was then verified through a joint evaluation by an oncology clinical treatment expert, two specialized oncology nurses, and six patient representatives.

## 3. Results

### 3.1. Characteristics of Participants

A total of 18 participants took part in the study, resulting in 20 interviews conducted. Participants 5 and 7 were each interviewed twice. The participants were 8 women and 10 men aged between 34 and 75 (mean 57.9 years). Their highest level of education was primary school or below (*N* = 6), junior high school or high school (*N* = 7), or university or above (*N* = 5). Geographically, ten participants came from rural areas, while eight were from urban areas. Employment status showed that three participants were employed, while fifteen were either retired or unemployed. The participants’ disease statuses were categorized as follows: four were in the maintenance therapy phase, nine were in the disease progression phase, and five were in the terminal phase. Regarding molecular pathological typing, there were seven cases each of EGFR mutation type and ALK rearrangement type, along with four cases of ROS1 rearrangement type. Among them, six were from the oncology department, three from the respiratory department, and nine from the radiotherapy department (Table 2).

### 3.2. Experience of Patients with Advanced NSCLC

**Figure 1:**
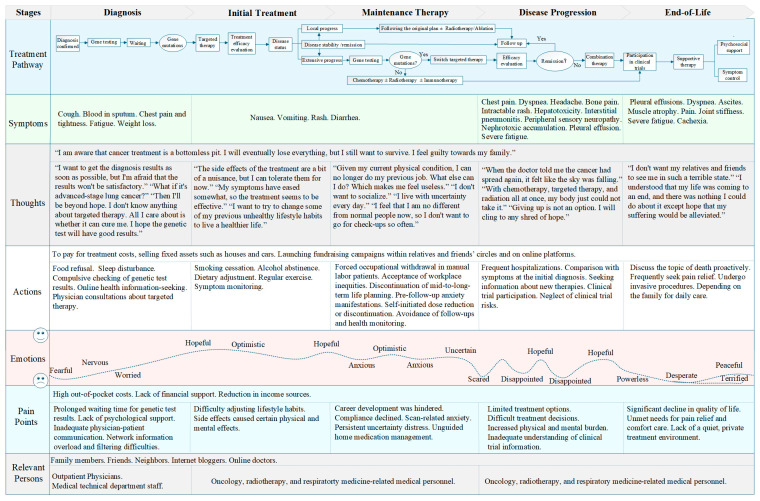
Journey map of patients with advanced NSCLC receiving targeted therapy.

This study divides the journey map of advanced NSCLC patients receiving targeted therapy into five stages along the horizontal axis: diagnosis, initial treatment, maintenance therapy, disease progression, and end-of-life. The vertical axis includes seven dimensions: treatment pathway, symptoms, thoughts, actions, emotions, pain points, and relevant personnel (Figure 1).

**Figure 2:**
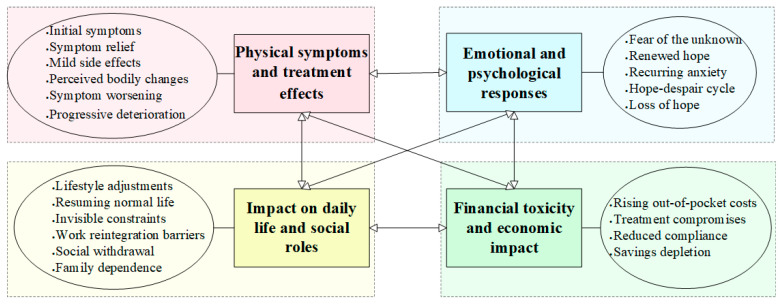
Patients’ experiences with advanced NSCLC receiving targeted therapy.

We developed four key themes: physical symptoms and treatment effects, emotional and psychological responses, impact on daily life and social roles, and financial toxicity and economic impact. Although presented individually, they are profoundly interconnected and often overlap throughout the patient journey, shaping a complex and multifaceted experience of targeted therapy for advanced NSCLC (Figure 2).

Theme 1: Physical symptoms and treatment effects

Participants reported that the initial signs of their lung cancer mainly included a persistent cough, hemoptysis, and chest pain. These symptoms often cause significant worry or fear: “It started with just a slight cough, but when I saw blood streaks in my phlegm, I became frightened.” (Participant 7).

After undergoing several cycles of targeted therapy, most participants noticed tangible physical improvements. The gradual alleviation of their original symptoms brought them considerable relief: “I can feel my breathing has become much easier, and it makes me feel so much relieved.” (Participant 9).

Although some participants reported experiencing side effects, mainly diarrhea, fatigue, and skin itching, these were mild and did not significantly affect their daily lives: “The diarrhea was certainly uncomfortable, but I can still go to work.” (Participant 6).

Once their disease stabilized, scan intervals for most patients were extended from every three months to six months or longer. The ongoing demands of managing a long-term illness heightened patients’ sensitivity to subtle bodily changes. As a result, participants often actively recognized signs of tumor recurrence or metastasis between scheduled scans, noticing the return of familiar symptoms. The return of these familiar symptoms usually caused significant anxiety, leading to earlier medical consultation: “My next check-up was not due for another three months, but I have been short of breath and had a headache for the past two weeks. I went to the hospital, and sure enough, it has spread.” (Participant 7).

As their conditions progressed, participants usually shifted from single-agent maintenance therapy to other targeted drugs, eventually moving to combination treatments that included chemotherapy, radiotherapy, and immunotherapy. Many participants reported a notable rise in the frequency and severity of side effects from using multiple medications together. Unbearable side effects, such as frequent nausea and vomiting, severe rashes, and cancer-related pain, caused great suffering and despair: “I cannot even keep down soup right now, I am just throwing up constantly. After a few rounds of radiotherapy, my neck is red, swollen, and burning with pain. This is the most painful thing I have ever experienced in my life.” (Participant 11).

The progression of functional decline and uncontrollable symptoms at the end-of-life stage led many participants to experience a deepening sense of helplessness and a loss of control over their own bodies: “I know the medical team did their best and threw everything they had at it, but I still cannot escape that suffocating feeling and the relentless pain that just will not leave me alone. I would rather just be dead.” (Participant 1).

Theme 2: Emotional and psychological responses

The initial 2-3 week wait for genetic test results marked a critical point, often described by many as “living under the sword of Damocles.” This period was characterized by dual uncertainty: worry about the lack of actionable genetic targets and fears of disease worsening during the wait: “The genetic testing process was an absolute nightmare. What if I do not qualify for treatment? What if my condition worsens again? I worry about it every single day.” (Participant 5).

After confirming an actionable therapeutic target, most participants experienced a significant positive shift in their emotions. This initial feeling of hope and optimism soon faded back into previous anxieties. However, these positive feelings were strengthened over time as symptoms improved and quality of life increased: “I am truly fortunate to have this drug. It has given me hope and a real chance at living a longer life.” (Participant 5).

Following a sustained positive response to treatment, some patients began maintenance therapy despite consistently receiving positive scan results. Most still experienced ongoing anxiety about the possibility of drug resistance, which was marked by mild fluctuations. This anxiety often appeared as a temporary feeling of relief after normal check-up results, only to return as the following scan approached: “Every time I see that my test results are normal, I feel a huge sense of relief. But the moment I think about the next check-up, the anxiety kicks in again.” (Participant 8).

When scan results confirmed tumor recurrence or metastasis, participants experienced a significant emotional decline, characterized by intense fear and despair: “When the doctor told me the cancer had metastasized again, I felt like my world was falling apart.” (Participant 13). Although adjustments to the treatment plan could provide a temporary emotional lift, the severe suffering from adverse side effects and the subsequent interruption in treatment often caused patients to revert to despair within weeks or even days. This created rapid cycles of hope and despair: “After developing resistance, the doctor said there was a new targeted drug I could try. I was only happy for a few days… The side effects were just too severe, and I instantly felt like I had fallen from the mountaintop into the depths.” (Participant 12).

After experiencing several therapeutic failures, participants increasingly lost faith in the treatment’s effectiveness. Having endured long-term suffering, most individuals in the terminal stage reached a point of resigned acceptance of death, with some even viewing it as a possible release from their pain: “For me, death would also be a kind of release; at least I would not have to suffer like this anymore.” (Participant 1). However, a few interviewees expressed fear of death: “I have no idea what is waiting for me after I die. Will I just… vanish into nothing? That is what terrifies me.” (Participant 16).

Theme 3: Impact on daily life and social roles

Beginning targeted therapy often served as a turning point, prompting patients to abandon harmful habits, such as smoking and drinking, and to pursue healthier routines, including improved diets and increased physical activity. Participants hoped these changes would boost the effectiveness of their treatment. However, changing habits built over many years was difficult, and family support was essential in this process: “After smoking for decades, quitting was hard. My wife kept an eye on me every day, and on the fourth try, I finally succeeded.” (Participant 8).

Benefiting from the favorable efficacy and low-toxicity nature of targeted therapies, most participants were able to return to their pre-treatment routines once their disease stabilized, reclaiming family responsibilities and re-engaging in social roles. For patients, it was a powerful affirmation of their self-worth and a restoration of dignity: “I still take my grandson to and from school every day, just like I used to. If I do not say anything, no one would even know I am sick.” (Participant 13).

For many patients, living with cancer remains a constant psychological burden. Fear of recurrence often causes some to withdraw from work and limit their social interactions. Additionally, overprotective behaviors from family members often contributed to patients’ feelings of inadequacy and reduced autonomy: “I am constantly terrified my cancer will come back. It has gotten so bad that I cannot bring myself to go to work or see my friends. My family is even more anxious—they watch me around the clock, doing everything for me. But this “care” makes me feel like a total burden, suffocating me with their overprotectiveness. I cannot breathe under all this pressure.” (Participant 9).

Some participants also mentioned obstacles such as promotion delays, job reassignments, and pay cuts due to employers’ concerns about their health: “After I got back to work, my boss always thinks I am not tough enough for the heavy lifting, so they keep me out of all the important projects.” (Participant 8).

Treatment-induced alopecia, significant weight loss, and skin reactions from combination therapy regimens left some participants experiencing profound self-consciousness. This often led to fewer visits from family and friends, gradually causing them to withdraw from their social networks: “I am so thin now, and I do not want anyone to see me. I am afraid they will stare at me with that stare.” (Participant 1).

After experiencing several episodes of disease progression, ongoing physical decline left many patients bedbound for long periods. Their reliance on others for basic daily activities such as eating, washing, and dressing often fostered a profound sense of lost dignity: “I am reduced to being completely dependent on my family, unable even to dress myself.” (Participant 10).

Theme 4: Financial toxicity and economic impact

After diagnosis, patients usually need targeted therapy until side effects become intolerable. Participants shared that, besides fixed medical expenses, such as medication, tests, and transportation, unexpected costs from managing complications such as rashes, diarrhea, and vomiting cause ongoing increases in out-of-pocket expenses: “The medication cost every month is already a huge burden on its own. Then, if my platelets suddenly drop, I need tests, maybe even injections, and trips to the hospital… and it is a constant financial worry.” (Participant 13).

Severe economic pressure led patients to constantly weigh survival benefits against treatment risks, ultimately resulting in compromises and choosing to participate in clinical trials: “I know this drug has its risks, but when it is a choice between life and death, you only have one option.” (Participant 6).

To cut costs, some participants engaged in treatment non-adherence behaviors such as voluntarily reducing medicine doses, discontinuing treatment, and skipping follow-up appointments. These actions often lead to higher overall expenses for future treatment and a greater symptom burden: “I was trying to cut corners by breaking my pills in half to save money. But when I saw the doctor again, he said things had taken a turn for the worse. Not only did this treatment end up costing more, but my body had to endure so much more as well.” (Participant 3).

In the final stage of the disease, while costs for targeted therapies and frequent medical tests may decrease, expenses for hiring caregivers, buying specialized care supplies, and nutritional support remain crucial. For some families, financial reserves had already been depleted, leaving them with no choice but to sell property or to borrow money from relatives and friends. This often causes participants to feel intense guilt toward their families: “Sometimes I wonder, am I being too selfish? To prolong my own life for a few more days, and in the process, drag my whole family down with me.” (Participant 7).

## 4. Discussion

This study described the journey of patients with advanced NSCLC who received targeted therapy. Our findings indicated that a continuous and dynamic interaction among physical symptoms, emotional and psychological responses, daily life and social roles, and financial toxicity collectively shapes the unique treatment experience of cancer patients.

Physical symptoms and treatment effects are fundamental to understanding patients’ experiences with targeted therapy for advanced NSCLC. These bodily changes not only affect patients’ functional abilities but also shape their emotional responses and their capacity to adapt to daily disruptions. Existing studies show that 50% of advanced lung cancer patients experience more than 13 symptoms [21]. The severity of functional impairment caused by these symptoms, together with the accompanying psychological distress, reflects the complexity of symptom burden [22], particularly for subjective symptoms such as anorexia, sleep disturbances, and pain, which are not easily identified through laboratory tests [23]. High symptom burden has been associated with a higher incidence of depression and anxiety, which in turn can exacerbate physical symptoms such as fatigue, insomnia, and pain, creating a vicious cycle that severely compromises quality of life [24]. As a result, there is growing emphasis on psychological interventions to support symptom management [25]. For example, a systematic review has shown that incorporating mindfulness techniques into routine cancer care, alongside stress reduction, oncology rehabilitation, and cognitive behavioral therapy (CBT), can effectively and sustainably reduce symptoms such as depression, anxiety, and fatigue in cancer patients [26]. These findings highlight the need for multidisciplinary approaches that integrate psychosocial support and leverage social resources to alleviate symptom burden and improve clinical outcomes in patients undergoing targeted therapy.

This study revealed an important phenomenon: tumor recurrence or metastasis was often first identified by patients themselves, rather than through scheduled imaging exams. This reflects a shift in responsibility under the home-based oral targeted therapy model, where patients are required to actively participate in symptom monitoring and management [6]. According to Symptom Management Theory, symptom outcomes are shaped by the patient’s experience and the strategies used to manage those symptoms [27]. In this context, patients do not passively await clinical evaluation. Instead, they accumulate experiential knowledge through ongoing self-management and use it to interpret subtle bodily cues, such as chest pain, hemoptysis, or dyspnea, as potential signs of disease progression. This process demonstrates a form of agency and self-directed care, where patients become key decision-makers in initiating diagnostic re-evaluation and treatment adaptation.

Throughout this journey, patients’ psychological and emotional states display a distinctive pattern of “cyclical fluctuation,” which remains poorly described in the existing literature [28]. The waiting period for genetic testing results marks the first critical point of emotional fluctuation. Common emotions reported by patients during this time include anxiety, fear, and apprehension. With a median waiting time of 23 days from sample collection to report availability, this psychological pressure causes some patients to opt for platinum-based chemotherapy prematurely [29]. As a result, they may miss out on opportunities for targeted therapies or immunotherapy while facing risks of ineffective treatment and related side effects [30]

Following treatment initiation, two distinct emotional cycles emerge. The first cycle correlates with scheduled imaging assessments and is mainly characterized by recurring anxiety, although overall emotional fluctuations remain relatively mild. We propose that this pattern may stem from two primary factors. First, the inherent treatment resistance of targeted therapy creates uncertainty about survival benefits, fueling a persistent underlying fear of disease recurrence. Second, effective treatment with minimal side effects sustains patients’ hope for survival. This dual dynamic allows patients to stay optimistic about the future while still feeling anxiety related to their treatment.

The second phase of the emotional cycle corresponds with the progression of the disease, during which patients primarily experience alternating periods of intense despair and disappointment. This phenomenon may relate to patients holding exaggerated expectations about treatment effectiveness, including unrealistic hopes of achieving a complete cancer cure [31]. Additionally, the physical discomfort caused by either the cancer itself or the side effects of treatment can cause many patients to feel that life lacks hope or meaning [6]. This psychological state may explain why most patients ultimately accept their mortality in a measured way. Our findings suggest the need for stage-specific psychosocial interventions tailored to patients’ evolving emotional responses throughout the disease trajectory. Attending to these psychological shifts is essential for delivering compassionate and personalized care that supports not just survival, but also the emotional well-being and sense of self of patients with advanced disease.

Our findings show that cancer diagnosis often initiates a complex and dynamic process of role adjustment for patients, which is not simply a unidirectional decline but involves ongoing adaptation and coping. Early in the treatment process, many patients actively modify their lifestyles and regain a degree of independence and self-care, resuming family and social responsibilities. However, as the disease progresses, physical limitations increase, leading to a gradual loss of independence and reduced social participation, with greater reliance on family and caregivers [32]. These evolving changes in roles and functions can deeply affect patients’ identity and psychological well-being, potentially causing existential distress and challenges to their sense of meaning [33]. While cultural factors shape these experiences, the transition from independence to dependence is a universal challenge faced by cancer patients globally [34]. Additionally, cancer patients’ ability to adapt to living with cancer greatly influences treatment outcomes [35]. Therefore, it is crucial to incorporate key support networks, including family, friends, and community resources, into care plans for patients with NSCLC. At the same time, improving patient education to boost personal agency and promote proactive social support seeking is recommended.

Financial toxicity remains an ongoing challenge for patients throughout cancer treatment [36]. In China, lung cancer patients face out-of-pocket medical costs that can reach up to 60%. Specific cancer-related diagnostics, such as PET-CT scans and genetic testing, along with effective targeted therapies, are often not covered by basic medical insurance [37]. Previous studies show that severe financial hardship is associated with decreased treatment adherence and changes in therapeutic decisions, like postponing, forgoing, or stopping treatment [38,39]. These decisions frequently increase symptom burden and negatively affect patients’ physical, emotional, social, and functional well-being [40].

Our research found that patients primarily alleviate financial strain by using personal savings, borrowing money, or selling property, with limited outside support. In this context, financial navigation emerges as an effective way to help reduce financial toxicity [41]. By conducting structured and thorough assessments of financial toxicity risk factors, financial navigators can help access resources that meet financial needs during cancer care [25]. Randomized controlled trials have shown that financial navigation not only lowers medical costs but also significantly improves depression, anxiety, cancer-related distress, and perceived social support [42,43,44]. However, implementing such a service poses practical challenges, especially in resource-limited settings where dedicated financial navigator roles may not yet exist [45]. In these contexts, it may be necessary to integrate basic financial navigation functions into the roles of social workers, nurses, patient coordinators, or other healthcare professionals. At the same time, the establishment of formal financial navigator positions should be considered as part of a broader policy initiative. Building such capacity will require institutional commitment and cross-sector collaboration but may ultimately reduce treatment discontinuation and improve patient outcomes in advanced cancer care.

## 5. Strengths and Limitations

Patients were recruited for this study through purposive sampling in the departments of medical oncology, respiratory medicine, and radiation oncology to ensure a diverse and representative sample. During data collection, a multi-channel approach that combined qualitative interviews, patient journey logs, and electronic medical records enriched the data. The results were presented as patient journey maps that visually illustrated the dynamic changes in patient experiences. However, due to the unique characteristics of China’s healthcare system and culture, the study outcomes might not reflect the experiences of patients receiving targeted therapy for NSCLC in other countries. Additionally, since most participants were recruited during disease progression or end-of-life stages after extended illness periods, their memories of earlier experiences could be affected by recall bias.

## 6. Conclusions

Patients with advanced NSCLC face numerous challenges during targeted therapy. The journey map visually presents the patient’s experience from diagnosis to end-of-life stages, enabling healthcare professionals to understand the core needs of patients at each stage from a comprehensive perspective. Future care plans should be developed around key milestones such as genetic testing, treatment adjustments, and disease progression, addressing patients’ physiological and psychological challenges at different stages to align care interventions with patient needs.

## Figures and Tables

**Table 1:** Interview guide.

Main Topics	Questions
Experiences of advanced non-small cell lung cancer patients with targeted therapy	1. Can you describe your experience from diagnosis to the beginning of treatment? Which parts had the most profound impact on you?
	2. Before starting treatment, what did you know about targeted therapy?
	3. During the initial stages of targeted therapy, did you have any specific feelings or experiences?
	4. How has long-term targeted therapy impacted your work, daily life, and family?
	5. How did you feel when your treatment plan needed adjustments?
	6. How smoothly did your transition to the new treatment plan go? What effects has the latest approach had on you?
	7. What led you to participate in a clinical trial?
	8. How has the progression of your illness physically challenged you? How do you view your current condition?

**Table 2:** Characteristics of participants (*N* = 18).

Variables	Groups	*N* (%)
Gender		
	Female	8 (44.4%)
	Male	10 (55.6%)
Age, years		
	30–45	2 (11.1%)
	46–60	10 (55.6%)
	61–75	6 (33.3%)
Educational Level		
	Primary or below	6 (33.3%)
	Junior or senior high school	7(38.9%)
	College or above	5 (27.8%)
Employment Status		
	Employed	3 (16.7%)
	Unemployed	15 (83.3%)
Place Residence		
	Rural	10 (55.6%)
	Urban	8 (44.4%)
Driver Genes		
	ROS1 rearrangement	4 (22.2%)
	EGFR mutation	7 (38.9%)
	ALK rearrangement	7 (38.9%)
Department		
	Medical oncology	6 (33.3%)
	Respiratory medicine	3 (16.7%)
	Radiation oncology	9 (50.0%)
Treatment Stage		
	Maintenance therapy	4 (22.2%)
	Disease progression	9(50.0%)
	End-of-life	5 (27.8%)

Abbreviations: EGFR, epidermal growth factor receptor; ALK, anaplastic lymphoma kinase; ROS1, ROS proto-oncogene 1, receptor tyrosine kinase.

## Data Availability

Data are contained within the article.

## Supplementary Materials

The following supporting information can be downloaded at: https://www.mdpi.com/article/10.3390/curroncol32080451/s1. File S1: Patient journey log. File S2: SRQR checklist. File S3: Interview guide.

## Abbreviations

The following abbreviations are used in this manuscript:

NSCLC	Non-small Cell Lung Cancer
EGFR	Epidermal Growth Factor Receptor
ALK	Anaplastic Lymphoma Kinase
ROS1	ROS proto-oncogene 1, receptor tyrosine kinase.
