# Supplementary material for: Experiences of Advanced Non-Small Cell Lung Cancer Patients with Targeted Therapy Using Journey Mapping: A Qualitative Study
Source: Curr Oncol. 2025 Aug 11;32(8):451. doi: 10.3390/curroncol32080451 (PMC12384887; doi:10.3390/curroncol32080451)
Supplement: Supplementary file 1 [file curroncol-32-00451-s001.zip › Interview Guide(File S3).pdf]

Interview Guide

This interview guide is designed for advanced non-small cell lung cancer patients receiving targeted therapy in this study. During the interview, the interviewer will adjust questions based on the patient's disease stage.

1. Can you describe your experience from diagnosis to the beginning of treatment? Which parts had the most profound impact on you?

2. Before starting treatment, what did you know about targeted therapy?

3. During the initial stages of targeted therapy, did you have any specific feelings or experiences?

4. How has long-term targeted therapy impacted your work, daily life, and family?

5. How did you feel when your treatment plan needed adjustments?

6. How smoothly did your transition to the new treatment plan go? What effects has the latest approach had on you?

7. What led you to participate in a clinical trial? Were you completely informed about its details?

8. How has the progression of your illness physically challenged you? How do you view your current condition?

9. What advice would you give to patients starting targeted therapy?
