# Supplementary material for: Experiences of Advanced Non-Small Cell Lung Cancer Patients with Targeted Therapy Using Journey Mapping: A Qualitative Study
Source: Curr Oncol. 2025 Aug 11;32(8):451. doi: 10.3390/curroncol32080451 (PMC12384887; doi:10.3390/curroncol32080451)
Supplement: Supplementary file 1 [file curroncol-32-00451-s001.zip › Patient Journey Log(File S1).pdf]

My Journey Log

Date:

Today’s Symptoms

Fatigue □None □Mild □Moderate □Severe

Pain □None □Mild □Moderate □Severe

Vomiting □None □1-2 times

□3-5 times □ ≥ 6 times

Diarrhoea □None □1-4 times

□4-6 times □>7 times

Other symptoms: ____________________

Medical Procedures

Blood tests □

Imaging studies □

IV infusion □

Oxygen therapy □

Thoracentesis □

Other: ____________________

Key Contacts

Doctor □

Nurse □

Family member □

Fellow patient □

Other: ____________________

Topics discussed: ____________________

Free Writing Space

Please write down anything you'd like to record:

Mood & Emotions

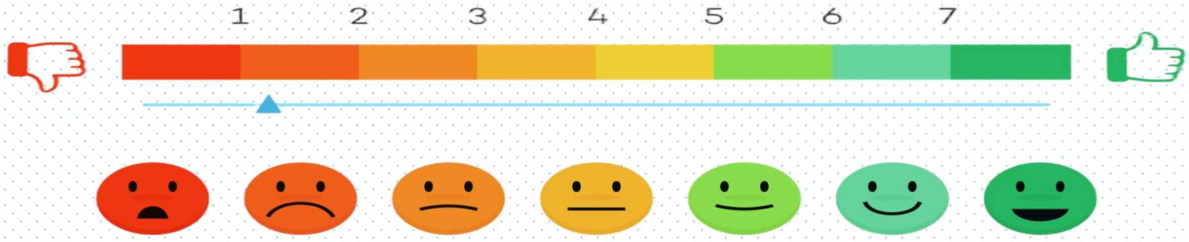

Other: ____________________
